# Joining forces: Leveraging novel combination therapies to combat infections with eukaryotic pathogens

**DOI:** 10.1371/journal.ppat.1009081

**Published:** 2020-12-31

**Authors:** Rachel E. Ham, Lesly A. Temesvari

**Affiliations:** 1 Department of Biological Sciences, Clemson University, Clemson, South Carolina, Unites States of America; 2 Eukaryotic Pathogens Innovation Center (EPIC), Clemson University, Clemson, South Carolina, Unites States of America; University of Wisconsin Medical School, UNITED STATES

## Introduction

Rising drug resistance, in pathogenic parasites and fungi, has become a major public health threat (reviewed in [1]). In endemic areas, substantial morbidity and mortality are caused by drug-resistant (DR) species. Resistant pathogens may also complicate treatments for noncommunicable conditions such as cancer chemotherapy and organ transplantation. The problem of drug-resistance is heightened by a lack of investment in novel antibiotic drug discovery by the pharmaceutical industry, in part, because there is a low rate of return for such drugs, compared with those used to treat chronic illnesses. While the development of new antimicrobial drugs is one approach in countering the DR phenotype, the discovery of useful drug combinations represents an alternative tactic that may expedite alternate clinical treatments.

**Fig 1:**
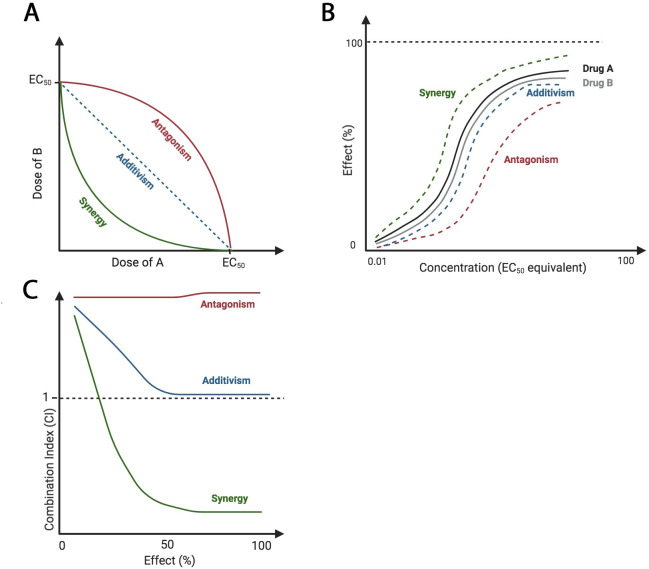
Theoretical isobologram, curve shift, and combination index analyses of drug–drug interactions. Antagonistic relationships require increased dosage of drugs for the same individual effects, additive effects require the same dosage of drugs for the same individual effects, and synergistic interactions require decreased dosage of drugs for the same individual effects. (A) Isoboles connect the points of EC_50_ of each drug, A and B. If the drugs are synergistic, the EC_50_ of agent A in the presence of B should be lower than that of agent A alone. (B) Curve shift analysis reveals a leftward shift, indicating synergy, or a rightward shift, indicating antagonism. This corresponds to changes of EC_50_. (C) CI values less than, equal to, or greater than 1 indicate synergy, additivism, or antagonism, respectively. Images created with BioRender.com. CI, combination index; EC_50_, half maximal effective concentration.

Using 2 or more drugs simultaneously does not guarantee that efficacy will exceed single-agent treatments. Pairwise drug interactions can be classified as either antagonistic, additive, or synergistic. Antagonism occurs when 2 drugs inhibit the therapeutic effects of each other. Additivism occurs when 2 drugs cause the combined predicted effect for both individual drugs. Synergism occurs when the combination of drugs exceeds the predicted effect of the combination [2]. Additionally, an inert agent, without an individual effect, can potentiate the action of a second drug [3]. Combination treatment regimens are often determined empirically but are based on a mathematical understanding of pairwise relationships, which is often calculated through isobologram, combination index, and/or curve shift analyses (Fig 1). In designing combination therapies, synergism is an important goal as it has the highest potential to lower off-target effects by decreasing doses (synergistic potency) and to improve clinical outcome by increasing desired effect (synergistic efficacy) [4].

Many combination therapies are used clinically to treat infections caused by eukaryotic pathogens, including giardiasis, cryptococcal meningitis, malaria, and trypanosomiasis [5,6]. In particular, combinations of either 2 artemisnin-derived compounds or atovaquone with proguanil have been instrumental in combatting DR malaria strains in endemic areas [7]. Furthermore, the trypanosomiasis combination therapy, elflornithine and nifurtimox, required only 8 years of development to reach clinical approval, which demonstrates the potential for rapid treatment development from combining drugs [8]. In light of this, the purpose of this article is to highlight advances (≤5 years) in identifying new combination therapies for DR parasites and fungi. Table 1 summarizes the combinations discussed and includes information about whether the combinations were found to be additive (+) or synergistic (*) and whether the combinations were assessed *in vitro* (^a^), *in vivo* in animal models (^b^), or in human patients (^c^). Because the combinations reviewed here have been newly discovered, the majority have not yet been tested clinically.

**Table 1:** Additive and synergistic drug combination strategies assessed against multiple eukaryotic pathogens.

	Combination 1	Combination 2	Combination 3	Combination 4	Combination 5
EXTRACELLULAR PATHOGENS					
Amoebozoa					
*Acanthamoeba castellanii*	^+^Simvastatin, voriconazole^a^ [23]	-	-	-	-
*Blastocystis* sp.	*Atorvastatin, metronidazole^b^ [26]	-	-	-	-
*Entamoeba histolytica*	*Simvastatin, lonafarnib^a^ [30]	*Lonafarnib, metronidazole^a^ [30]	-	-	-
*Naegleria fowleri*	*Auranofin, amphotericin B^a^ [53]	-	-	-	-
Diplomonads					
*Giardia intestinalis*	*Paromomycin, bacitracin^c^ [5]	*Nitroimidazoles, albendazole^c^ [16–18]	*Nitroimidazoles, paromomycin^c^ [16–18]	*Nitroimidazoles, mepacrine^c^ [16–18]	-
Fungi					
*Aspergillus fumigatus*	^+^Posaconazole, clofazamine^a^ [15]	^+^Caspofungin, clofazamine^a^ [15]	-	-	-
*Candida albicans*	^+^Fluconazole, clofazamine^a^ [15]	^+^Caspofungin, clofazamine^b^ [15]	*Amiodarone, caspofungin^a^ [15]	*Itraconazole, *Mentha* × *peperita* extracts^a^ [40]	-
*Cryptococcus neoformans*	^+^Fluconazole, amphotericin B^b^ [19]	*Amphotericin B, *Ocimum basilicum* extracts^a^ [42]	*Fluconazole, tomatidine^a^ [15]	*Itraconazole, *Mentha* × *peperita* extracts^a^ [40]	-
*Fonsecaea monophora*	*Terbinafine, amphotericin B^a^ [20]	-	-	-	-
Helminths					
*Echinococcus granulosus*	^+^Albendazole, 5-fluorouracil^b^ [36]	^+^Albendazole, *Zataria microflora* extracts^b^ [41]	^+^Albendazole, metformin^b^ [52]	^+^Albendazole, *Sophora moorcroftiana* alkaloids^b^ [48]	-
Trypanosomes					
*Trypanosoma brucei*	^+^Temozolomide, elflornithine^a^ [8]	^+^Temozolomide, melarsoprol^a^ [8]	-	-	-
*Trypanosoma congolense*	^*^Anogeissus leiocarpus, *Khaya senegalensis* extracts, potash^b^ [47]	-	-	-	-
INTRACELLULAR PATHOGENS					
Apicomplexans					
*Babesia* sp.	^+^Diminizene aceturate, clofazamine^b^ [11]	*Atovaquone, eflornithine^b^ [32]	^+^Diminizene aceturate, synthetic chalcones^b^ [38]	*Atovaquone, hydroxyurea^a^ [32]	-
*Plasmodium falciparum*	*B02, artemisinin^a^ [34]	^+^Prochlorperazine, chloroquine^a^ [60]	-	-	-
*Plasmodium vivax*	^+^Chlorpheniramine, mefloquine^b^ [59]	-	-	-	-
*Toxoplasma gondii*	^+^Clindamycin, azithromycin^c^ [9]	*Ketotifen, pyrimethamine^b^ [51]	^+^Simvastatin, pyrimethamine^a^ [27]	*Atorvastatin, C7S^b^ [25]	-
Trypanosomes					
*Leishmania amazonensis*	^+^Tamoxifen, amphotericin B^b^ [37]	-	-	-	-
*Leishmania donovani*	^+^Lovastatin, chromium chloride^a^ [28]	-	-	-	-
*Leishmania infantum*	*Nelfinavir, amphotericin B^b^ [49]	^+^Lopinavir, miltefosine^b^ [50]	*Sorafinib, auranofin, lopinivir^a^ [62]	-	-
*Leishmania major*	^+^Paromomycin, 4-aminoquinoline^b^ [14]	^*^Moringa oleifera extracts, amphotericin B^a^ [43]	-	-	-
*Leishmania martiniquensis*	*Allicin, amphotericin B^a^ [44]	-	-	-	-
*Leishmania mexicana*	^+^Paromomycin, 4-aminoquinoline^b^ [14]	-	-	-	-
*Trypanosoma cruzi*	*Clofazamine, benznidazole^a^ [12]	^+^*Chlamydomonas reinhardtii* extracts, nifurtimax^a^ [45]	*Clomipramine, benznidazole^b^ [58]	^+^Amiodarone, benznidazole^a^ [54]	*Lippia alba limonene and citral^a^ [46]

Combinations marked with (+) are additive, and combinations marked with (*) are synergistic.

^a^ Assessed in vitro.

^b^ Assessed *in vivo* in animal models.

^c^ Assessed *in vivo* in human patients.

### Conventional antimicrobial and antifungal agents—Better together

Combining conventional drugs that target eukaryotic pathogens has resulted in improved inhibition of pathogen growth. Furthermore, some antimicrobials that target prokaryotes have secondary antiparasitic targets and potentiate the action of standard antiprotozoal drugs. For example, neuroinvasive *Toxoplasma gondii* was successfully countered with a combination of clindamycin and azithromycin, both of which inhibit protein synthesis in bacteria. Although the antiparasitic mechanism is unclear, clindamycin effectively crosses the blood–brain barrier and accumulates in tissues with high parasitic loads [9].

Diminizene aceturate is a DNA-binding drug commonly used to treat canine babesiosis [10], but it has not been widely used in humans due to its narrow therapeutic window. However, combination therapy, using diminizene aceturate and clofazimine, an antimycobacterial drug, lowered the dose of diminizene aceturate that produced the same *in vivo* outcome as higher doses of diminizene aceturate in a mouse model of *Babesia* infection [11]. Both drugs bind to regions of DNA that possess a high content of adenine and thymine residues (AT-rich). AT-rich DNA is common in *Babesia* sp., and when constrained by the drugs, this DNA cannot be replicated, causing cell cycle arrest. Because clofazamine can lower the effective dose of diminizene aceturate, it may provide a larger therapeutic window for treating human babesiosis. Clofazimine also inhibits trypanosomal proline transporters and enhances retention of benznidazole, commonly used to treat *Trypanosoma cruzi* infections [12].

Paromomycin is the conventional treatment for cutaneous leishmaniasis. 4-Aminoquinoline chloroquine is often used in combination with doxycycline to treat Q fever, which is caused by intracellular infections with the bacterium, *Coxiella burnetii* [13]. Together, paromomycin and 4-aminoquinoline synergized in the treatment against *Leishmania major* and *L*. *mexicana* [14]. Both intracellular *Leishmania* amastigotes and *C*. *burnetii* bacteria induce acidification of macrophages, which decreases the therapeutic activity of conventional drugs. Therefore, alkalization of macrophages, by 4-aminoquinoline chloroquine, may have enhanced the cytotoxic mechanisms of doxycycline and paromomycin. Interestingly, clofazamine can also potentiate many antifungal drugs, including fluconazole, posaconazole, and caspofungin, to inhibit *Candida* sp. and *Aspergillus fumigatus* [15]. Clofazamine elicits cell membrane stress and activates protein kinase C-like 1 (Pkc1) signaling, which is also activated by fluconazole and caspofungin in *Candida albicans* [15]. Similarly, posaconazole and caspofungin each synergized with clofazimine to decrease the hyphal growth of *A*. *fumigatus* [15].

Combinations with 2 conventional antimicrobial agents have also been used to treat *Giardia intestinalis* infections. Successful combinations include paromomycin with bacitracin zinc, and nitroimidazole-derived drugs with either paromomycin, albendazole, or mepacrine. These combinations have been successful in human patients according to clinical reports and are extensively reviewed elsewhere [16–18]. However, these treatments have not yet been evaluated in formal clinical trials.

Combining multiple antifungals has also proven effective against many pathogens. Amphotericin B is used as a conventional treatment for several fungal infections. This drug interferes with ion transport and membrane polarization, leading to high host toxicity. Additionally, amphotericin B interferes with membrane permeability by binding to ergosterol, a pathogen-specific lipid that regulates outer membrane integrity. Fluconazole, a broad-spectrum antifungal, inhibits ergosterol synthesis; this drug synergized with amphotericin B by preventing the replacement of amphotericin B-bound ergosterol in *Cryptococcus neoformans* membranes [19]. Terbinafine, the conventional drug treatment for chromoblastomycosis, causes accumulation of an ergosterol intermediate that increases membrane permeability. Amphotericin B and terbinafine synergized through rapid membrane alterations, leading to enhanced lysis of melanized *Fonsecaea monophora*, one of the causes of chromoblastomycosis [20].

### Statins—Harnessing pleiotropic effects

Statins, such as simvastatin, atorvastatin, and lovastatin, are a class of drugs that inhibit 3-hydroxy-3-methyl-glutaryl-coenzyme A (HMG-CoA) reductase (HMGR) activity and are routinely used to treat hypercholesterolemia. HMGR catalyzes the biosynthesis of many sterols through the conversion of HMG-CoA into 1-mevalonate, a sterol precursor [21,22]. 1-Mevalonate also functions as a biochemical precursor of isoprenoid groups, which serve as posttranslational prenylations on many proteins. Statins are versatile because they have large therapeutic windows. Many parasites scavenge sterols from the host in addition to synthesizing sterols themselves. Therefore, combining multiple compounds that limit sterol synthesis in either pathogen or host is predicted to be synergistic.

Combining simvastatin with the antifungal drug, voriconazole, was effective in targeting 2 different biochemical steps in *Acanthamoeba* ergosterol synthesis [23]. Simvastatin blocked amebic HMG-CoA, while the antifungal drug voriconazole blocked 14α-demethylase, an enzyme responsible for assembling ergosterols [24]. In a mouse model of *T*. *gondii* infection, strong synergism was observed between atorvastatin and C7S, a bisphosphonate that also functions as an inhibitor of parasitic isoprenoid synthesis [25].

Statins have been shown to be particularly useful against intracellular parasites because the drugs can alter host cell membranes and modulate host immunity. Atorvastatin enhances host cellular membrane integrity during *Blastocystis* infection, preventing protozoan invasion and leaving parasites vulnerable to metronidazole [26]. Similarly, simvastatin decreased *T*. *gondii* tachyzoite adhesion and invasion, allowing a conventional anti-toxoplasmosis drug, pyrimethamine, to kill exposed parasites in the bloodstream [27]. Statins may enhance oxidative stress, part of the host immune response. For example, combining lovastatin with the antileishmanial, chromium chloride, produced 2 varieties of reactive oxygen species in parasitic cells, which induced apoptosis in *Leishmania donovani* amastigotes [28].

Statins can pleiotropically inhibit protein prenylation in a number of conserved protein families, including those in the Ras superfamily of G-proteins. Farnesyltransferase is an essential enzyme that catalyzes the posttranslational attachment of farnesyl groups on proteins. Combination therapy with simvastatin and lonafarnib, an antineoplastic that inhibits farnesyltransferase [29], was synergistic against *Entamoeba histolytica* [30]. This finding was unanticipated because genome data suggest that *E*. *histolytica* lacks HMGR. Thus, the therapeutic efficacy of simvastatin may be due to pleiotropic effects in the isoprenoid biosynthesis pathway. Combination therapy with the standard antiprotozoal, metronidazole, and lonafarnib was also synergistic against *E*. *histolytica* [30]. This outcome may have been due to lonafarnib-induced membrane destabilization, caused by impaired protein prenylation, which, in turn, intensified oxidative damage caused by metronidazole.

### Antineoplastics—DNA damage and membrane transport disruption

Many antineoplastic drugs inhibit unrestrained tumor cell proliferation by targeting DNA or DNA repair. Such drugs may potentiate conventional antimicrobials that function by damaging DNA. The antiparasitic drug, atovaquone, which induces double-stranded DNA breaks [31], synergized with either hydroxyurea or eflornithine, 2 antineoplastic agents, to block multiplication and differentiation of *Babesia* parasites [32]. *Trypanosoma brucei* growth was inhibited by combining the antineoplastic agent, temozolomide, with either eflornithine or melarsoprol, both standard medications used for treatment of sleeping sickness. Temozolomide, developed for glioblastoma, is a DNA-damaging agent that crosses the blood–brain barrier. Thus, it is particularly advantageous for cases with brain migration of trypanosomes [8]. Temozolomide was shown to trigger trypanosomal cell cycle arrest through DNA damage, limiting proliferation and increasing parasitic susceptibility to standard treatments. Similarly, B02, an inhibitor of DNA repair [33], synergistically enhanced the DNA damaging activity of artemisinin and chloroquine in *Plasmodium falciparum* [34]. *Echinococcus* cell division was limited by the DNA damaging antiparasitic drug, albendazole [35], and the antineoplastic, 5-fluorouracil, a nucleoside analog that is incorporated into DNA and produces toxic metabolites [36]. Tamoxifen, commonly used to inhibit human estrogen receptors, increases membrane depolarization in *Leishmania amazonensis* and may also depolarize mitochondrial membranes, leading to synergy with amphotericin B [37].

### Natural compounds—Expanding the toolbox of traditional medicine

Combination methodology is commonplace within traditional medicine because raw plant material contains bioactive compounds with unknown mechanisms. Chalcones are flavonoids that are plant-derived phenolic compounds. Synthetic chalcones inhibit glucose metabolism of *Babesia* and reduce the toxicity of diminizene aceturate through an unknown mechanism [38]. Tomatidine, an alkaloid originating from tomato (*Solanum lycopersicum*) that is conventionally used to inhibit skeletal muscle atrophy, synergized with fluconazole to reduce the growth of *C*. *albicans* [15]. This alkaloid inhibits sterol methyltransferase and sterol reductase, members of the ergosterol synthesis pathway in several fungal species [39]. Similarly, peppermint (*Mentha* × *peperita*) phenolic extracts inhibit ergosterol synthesis and increase intracellular acidification by blocking plasma membrane ATPases; these extracts synergized with itraconazole against *C*. *albicans* and *C*. *neoformans* [40]. Albendazole activates macrophages and impairs motility of *Echinococcus* cells, and both activities can be improved with *Zataria multiflora* phenolic extracts. *Z*. *multiflora* extracts increased membrane permeability and altered parasite motility [41].

Additionally, amphotericin B synergizes with many phenolic plant extracts to inhibit the growth of several pathogens. Two models have been proposed for phenolic extract synergy with amphotericin B: enhanced lipophilic transport that increases drug permeation and inhibition of outer membrane ergosterol exchange with intercellular membranes. For example, phenolic extracts from basil (*Ocimum basilicum*) synergized with amphotericin B to inhibit the growth of *C*. *neoformans* through increased lipophilic transport of drug [42]. Similarly, *Moringa oleifera*, a medicinal plant, also produced phenols that increased lipophilic transport of amphotericin B into *L*. *major* parasites [43]. The garlic-derived compound, allicin, inhibited outer membrane ergosterol exchange with vacuole membranes and increased the rate of amphotericin B interference with membrane ergosterol in *Leishmania martiniquensis* [44]. Phenolic compounds may also bind intracellular proteins and increase oxidative stress in *L*. *martiniquensis*, enhancing the antiparasitic activity of amphotericin B [44].

Other non-phenolic–derived compounds can enhance standard antimicrobial treatments. Microalgae extracts from *Chlamydomonas reinhardtii* inhibited intracellular invasion of *T*. *cruzi*, potentiating the action of nifurtimox, a conventional treatment only active against bloodstream trypomastigotes [45]. Alternatively, natural compounds may produce synergy through immune system stimulation and protection from drug-induced host toxicity. Benznidazole induces oxidative stress that damages both *T*. *cruzi* and host cells; however, supplementation with *Lippia alba*–derived terpenes inhibited host inflammation and may have induced additional parasite-specific oxidative stress [46]. *Trypanosoma congolense*, the major agent of cattle trypanosomosis that also serves as a model organism for human *T*. *brucei* infection, exhibited impaired motility in a mouse model of infection caused by a combination of extracts from *Anogeissus leiocarpus* and *Khaya senegalensis* along with potash salts, resulting in decreased parasitemia [47]. Albendazole treatment of *Echinococcus* infections can also be enhanced by a combination treatment with *Sophora moorcroftiana* alkaloids, which increase host complement activation [48].

### Surprisingly successful combinations—Repurposed drugs

Drug screening has revealed unexpected agents with antiparasitic activity. Furthermore, clinicians have observed improved outcomes from patients with protozoan infections already taking chemotherapeutics for unrelated conditions. Patients infected with *L*. *infantum* and taking nelfinavir, an antiretroviral for treatment of concurrent HIV, had better outcomes than HIV–negative patients taking only amphotericin B [49]. *L*. *infantum* infections were also cleared more quickly when treated with a combination of an antiviral, lopinavir, and an antileishmanial, miltefosine [50]. Upon further investigation, it was shown that both antiretrovirals prevented intracellular multiplication of amastigotes [49] and altered parasite membrane lipid composition [50], leading to a reduced parasite load and synergy with conventional antiparasitic drugs. Likewise, ketotifen, a tricyclic antihistamine, prevented host cell invasion by *T*. *gondii* through stabilization of host cell membrane. This drug also inhibits intracellular parasite multiplication, which increases the percentage of bloodstream parasites that are vulnerable to pyrimethamine [51]. Interestingly, ketotifen may also reduce host cell inflammation, rendering infections less harmful [51]. Finally, metformin, a hydrophilic antihyperglycemic that also prevents parasitic proliferation, improved conventional albendazole treatment of echinococcosis resulting in reduced parasite load [52].

Several repurposed drugs may increase the delivery of conventional drugs to target tissues. Auranofin, an anti-arthritic drug, synergized with conventional amphotericin B to inhibit growth of *Naegleria fowleri* [53]. Auranofin impairs the *N*. *fowleri* oxidative stress response by inhibiting both selenoprotein synthesis and thioreductase activity. It also may improve drug delivery of amphotericin B across the blood–brain barrier, which might be advantageous for treating neuroinvasive *N*. *fowleri* infections [53]. Conventional treatment of *T*. *cruzi* with benznidazole was enhanced with amiodarone, a cardiac antiarrhythmic drug often prescribed for symptomatic treatment of invasive cardiac cases of Chagas disease. Both drugs were shown to have distinct targets in *T*. *cruzi* and to reach therapeutic concentrations in myocardial tissue. Benznidazole caused oxidative stress and amiodarone inhibited parasitic ergosterol biosynthesis, and the combination decreased parasitic cell counts *in vitro* more effectively than single-drug treatment [54]. Amiodarone also impairs ion transport in *C*. *albicans* [55] and has synergy with caspofungin inhibition of β(1,3)-glucan synthase, leading to decreased cell wall integrity and limited fungal growth [15].

Many repurposed drugs also exhibit binding to parasite-specific proteins. Clomipramine, a tricyclic antidepressant, binds trypanothione reductases and mitochondrial membranes in many trypanosomatida, including *L*. *amazonensis* [56], *T*. *brucei* [57], and *T*. *cruzi* [58]. Trypanothione reductase is a protein that is critical in the antioxidant response of *T*. *cruzi*; thus, oxidative stress, inflicted by benznidazole, may be intensified by an inhibited antioxidant response mediated by clomipramine [58]. The antipsychotic drug, prochlorperazine [59], and the antihistamine, chlorpheniramine [60], both potentiated chloroquine and mefloquine by binding parasite-specific efflux pumps and ATP-binding cassette (ABC) transporters in *Plasmodium vivax* [59] or *P*. *falciparum* [60]. This, in turn, forced the retention of the conventional antimalarial drugs.

### Future perspectives—Nearly endless combinations

Combination therapies have been reported for most infections with eukaryotic pathogens. An abundance of studies in the last 5 years demonstrates that there are many viable avenues including those that use antibiotic/antibiotic combinations and antibiotic/nonantibiotic combinations. Notably, such combination regimens are not without risk. Potential disadvantages include the possibility of increased side effects coupled with a difficulty in discerning the source of side effects, increased toxicity, incompatible pharmacokinetics, and increased cost [7,61]. Conversely, many of these shortcomings could be offset by the potential that combination therapies may result in lowering the effective dosage of each drug in the combination and/or shortening drug treatment regimens.

Capitalizing on the wide availability of data and the variety of drug mechanisms may also yield promising 3-way combinations in the future. For example, *L*. *infantum* parasites have been more successfully eliminated with a combination of sorafinib, a kinase inhibitor used to treat kidney cancer, lopinavir, an antiretroviral, and auranofin, an inflammation reducing anti-arthritic, than when treated with these agents alone [62]. Pairwise combinations of these 3 drugs were additive, but the combination of all 3 produced synergy. Furthermore, the complete cure of DR giardiasis, using paromomycin, albendazole, and metronidazole, has been reported [17]. Overall, the lack of development of novel drugs, the consistent resistance acquisition caused by drug monotherapies, and the preliminary success of many of the approaches described here makes the combination approach vital in the war against DR eukaryotic pathogens.
